# A new method for pharmaceutical compounding and storage of anti-VEGF biologics for intravitreal use in silicone oil-free prefilled plastic syringes

**DOI:** 10.1038/s41598-019-54226-7

**Published:** 2019-12-02

**Authors:** Heidrun Elisabeth Lode, Torleif Tollefsrud Gjølberg, Stian Foss, Magne Sand Sivertsen, Jørgen Brustugun, Yvonne Andersson, Øystein Kalsnes Jørstad, Morten Carstens Moe, Jan Terje Andersen

**Affiliations:** 1 Department of Immunology, Oslo University Hospital Rikshospitalet, Oslo, Norway; 2 Institute of Clinical Medicine and Department of Pharmacology, University of Oslo and Oslo University Hospital, Oslo, Norway; 3 Department of Ophthalmology, Oslo University Hospital and Faculty of Medicine, University of Oslo, Oslo, Norway; 4 Department of Biosciences, University of Oslo, Oslo, Norway; 5 Hospital Pharmacies Enterprise, South-Eastern Norway Regional Health Authority, Oslo, Norway

**Keywords:** Therapeutics, Proteins

## Abstract

Intravitreal injections of antibody-based biologics targeting vascular endothelial growth factor (VEGF) are highly effective and have markedly decreased the risk of visual impairment associated with prevalent retinal diseases, such as neovascular age-related macular degeneration and diabetes macular oedema. The diseases are chronic in their nature, and most patients need long-term therapy to suppress disease activity. We previously reported a compounding method for repackaging and storage of aflibercept (Eylea), a commonly used anti-VEGF biologic, in silicone oil-coated plastic syringes without compromising drug stability or activity. In addition to improving safety and time spent per patient, compounding of anti-VEGF biologics enables single-dose vials to be split into multiple syringes, thereby considerably reducing waste and drug expenses. However, symptomatic silicone oil droplets may deposit in the eye’s vitreous body after repetitive injections. To fully avoid this complication, we here report on a novel pharmaceutical compounding method using silicone oil-free syringes and a 33 G × 9 mm Low Dead Space Needle hub injection needle. We evaluate the method for three anti-VEGF biologics commonly used in ophthalmology: aflibercept, ranibizumab (Lucentis) and bevacizumab (Avastin). Our results show that compounding and storage for one week does not compromise the functional activity of the biologics and allows for safe and cost-effective compounding of anti-VEGF biologics for intravitreal injections in prefilled silicone oil-free syringes.

## Introduction

Biologics targeting vascular endothelial growth factor (anti-VEGF) have revolutionized the treatment of retinal diseases causing altered vascular permeability, such as diabetic macular oedema, retinal vein occlusion and the neovascular type of age-related macular degeneration (nAMD). These diseases are chronic in nature, and the aim of anti-VEGF treatment is not to cure the patients, but rather to suppress disease activity. The patients are usually in need of long-term monitoring and treatment, and the injections are typically given at monthly to trimonthly intervals^1–4^. Accordingly, intravitreal anti-VEGF treatment places a significant burden on the patients. Moreover, costly drugs and extensive follow-up can be prohibitive and puts a heavy strain on healthcare systems. The number of patients in need of treatment is expected to dramatically increase in the coming years due to new indications for anti-VEGF therapy and an aging population^5^. Although the intravitreal route of administration is considered to be safe, there is also an inevitable risk of surgical complications, the most devastating being bacterial endophthalmitis^6,7^. All efforts should thus be made to handle the medication and perform the intravitreal procedure as safe and effective as possible, without causing unnecessary waste of the expensive biologics.

An intravitreal injection procedure begins with the preparation of the drug for administration. For both anti-VEGF agents currently approved for intravitreal use, ranibizumab (Lucentis) and aflibercept (Eylea), the label recommendation includes measures to prevent contamination^8,9^. The top of the vial must be cleaned with an alcohol wipe and the vial content withdrawn into a sterile syringe through a filter needle, which is replaced with an injection needle. Since this preparation is intended to occur at the site of injection, typically an office, clean room, or operating theatre, the label approves of suboptimal aseptic conditions. Repetitive preparation of syringes is also a time-consuming practice for clinicians. Altogether, these disadvantages have encouraged the establishment of pharmaceutical compounding of prefilled syringes for intravitreal use. Yet, studies on compounding of antibody-based biologics have resulted in varying, and sometimes contradictory, results in regard to both safety and drug integrity^6,7,10–19^. In a previous study we demonstrated that compounding of aflibercept in prefilled commonly used insulin syringes had no negative effects on drug properties, even after storing the syringes for weeks^10^. Such a compounding procedure may not only improve patient safety but also increase the focus on the patient rather than on drug preparation. Finally, compounding reduces waste of biologics and saves considerable costs for the healthcare system^10,20^.

**Figure 1:**
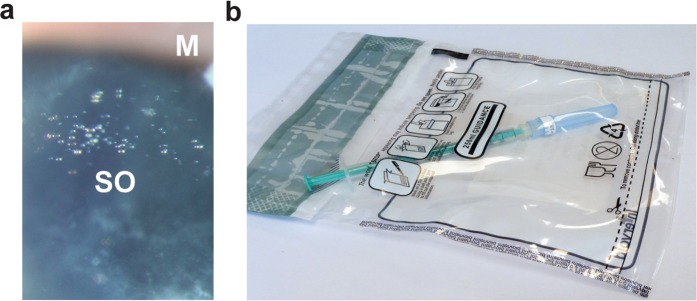
Eye with symptomatic silicone oil droplets and an anti-VEGF biologic after compounding. (**a**) Biomicroscopic picture of small silicone oil (SO) droplets in the upper anterior part of the vitreous body; the droplets appear as small clear spheres. Margo of upper eye lid (M). (**b**) Labelled plastic bag containing a prefilled, silicone oil-free plastic syringe attached to a capped needle. The images have been cropped.

Notably, most syringes used for intravitreal injections are coated with silicone oil which acts as a lubricant between the syringe barrel and plunger. Silicone oil may follow the drug intravitreally and lead to symptomatic deposition of silicone oil droplets (Fig. 1a), and there is an increasing concern about this particular adverse event. The use of syringes with low dead space^21–26^ and a practice of flicking the syringe before use^22^ have been shown to increase the risk. In pursuit of the safest possible intravitreal injection procedure, proper pharmaceutical compounding of anti-VEGF agents in pre-filled silicone oil-free syringes is warranted. Yet, as components of the pre-filled syringes could interfere with the biopharmaceuticals’ protein structures, an important consideration is that absence of a protective silicone oil layer might compromise drug structure and effectiveness.

The purpose of the present study was to establish a novel procedure for safe and cost-effective pharmaceutical compounding of pre-filled silicone oil-free syringes containing ranibizumab, aflibercept, or bevacizumab for intravitreal use and to investigate the structural integrity of the three anti-VEGF biologics after one week of storage.

## Results

### Compounding in silicone oil-free syringes

To establish a novel repackaging procedure for compounding of the anti-VEGF biologics in syringes without silicone oil-coating, we used 1 mL syringes that are both silicone oil-free and have a low dead space^22^. Drug withdrawal took place at the hospital pharmacy, utilizing an isolator unit with a class A production chamber and a class B transfer chamber. The syringes were aseptically filled with 0.06 mL ranibizumab (0.6 mg), aflibercept (2.4 mg) or bevacizumab (1.5 mg). The syringes were then attached to a Low Dead Space Needle hub 33 G × 9 mm injection needle intended for ophthalmic use. Each syringe with needle was separately enclosed in a sterile, transparent plastic bag (Fig. 1b) and stored at 4 °C and in dark conditions for 0 days (D0) or 7 days (D7). The volume retrieved at D7, when manually adjusting the plunger to 0.05 mL before fully depressing it, was 0.0494 ± 0.0060 mL (n = 10), i.e. 98.8% of the intended volume.

### Protein concentration and stability

**Figure 2:**
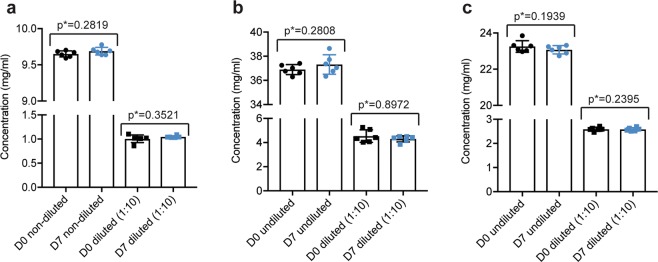
Concentrations of the three anti-VEGF biologics before and after storage in silicone oil-free plastic syringes. Undiluted and diluted (1:10) samples collected at day 0 (D0) shown in black, and day 7 (D7) shown in blue for (**a**) ranibizumab, (**b**) aflibercept and (**c**) bevacizumab. Measurements are presented as mean ± SD. For each sample set n = 6. The unpaired Student’s t-test was used for statistical analysis.

First, to compare the syringes’ D0 and D7 anti-VEGF concentrations, measurements were performed using a DeNovix DS-11+ Spectrophotometer. For the three biologics, D0 and D7 concentrations from both undiluted and diluted samples did not statistically differ (Fig. 2). Thus, the absence of silicone oil-coating did not affect adsorption of the biologics to the plastic of the syringes.

**Figure 3:**
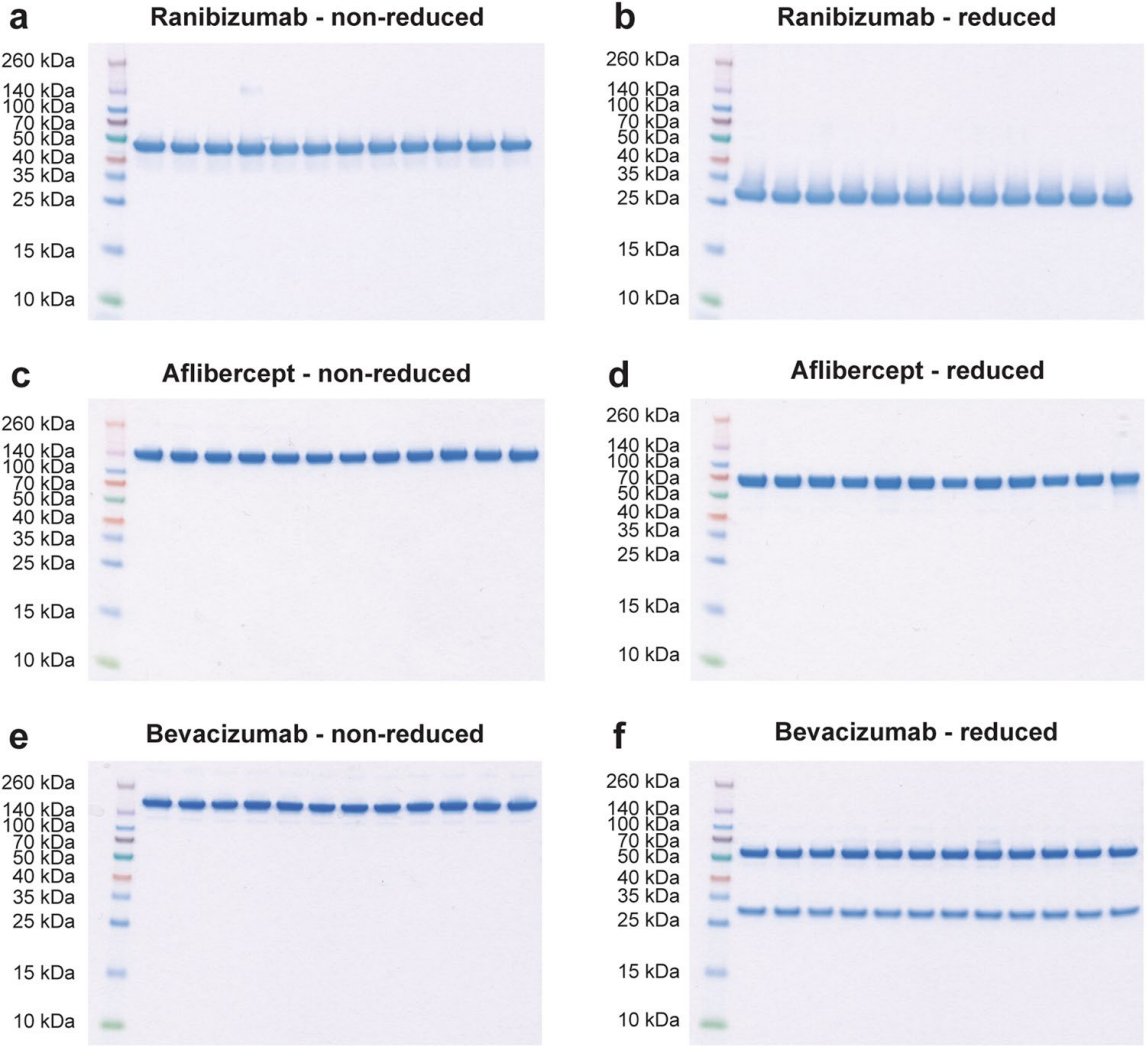
SDS-PAGE analysis. Non-reducing and reducing SDS-PAGE analysis of (**a**,**b**) ranibizumab D0 (sample 1–6) and D7 (sample 7–12), (**c**,**d**) bevacizumab D0 (sample 1–6) and D7 (7–12), and (**e**,**f**) ranibizumab D0 (sample 1–6) and D7 (sample 7–12). The images have not been cropped.

**Figure 4:**
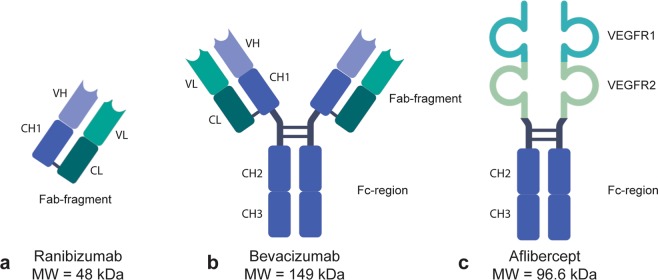
Schematic overview of three commercially available anti-VEGF biologics. Ranibizumab (**a**) is composed of the affinity maturated Fab of bevacizumab, (**b**) bevacizumab is a full-length IgG1 antibody and (**c**) aflibercept is composed of domains from VEGFR1 and 2 fused to an IgG1 Fc-region. The figure was created with BioRender software.

Secondly, to verify anti-VEGF integrity, equal amounts of each biologic were added to SDS-PAGE gels under reducing and non-reducing conditions. Inspection of the Coomassie-stained gels under non-reducing conditions revealed that the three biologics migrated as major bands of 50 kDa for ranibizumab and approximately 150–170 kDa for aflibercept and bevacizumab. Bevacizumab migrated somewhat slower than aflibercept. The migration profiles are in line with their expected molecular weights (Fig. 3). Ranibizumab, a Fab fragment, consists of a light chain (VL-CL) covalently connected to two heavy chain domains (VH-CH1), whereas bevacizumab, a full-length IgG1 antibody, consists of two light chains paired with two complete heavy chains (VH-CH1-CH2-CH3)^9^ (Fig. 4a,b). Aflibercept, a recombinant decoy receptor, is an IgG1 Fc-fusion where the extracellular domain 2 of VEGF receptor (VEGFR) 1 is genetically fused to domain 3 of VEGFR2 and connected to the Fc-region of human IgG1^27^ (Fig. 4c). Accordingly, under reducing conditions ranibizumab migrates as one major band of 25 kDa, whereas bevacizumab and aflibercept migrate as bands with a molecular weight of approximately 70–80 kDa. Bevacizumab additionally displayed a minor band, corresponding to a light chain of 25 kDa. Importantly, the biologics exhibited no visible changes in integrity between D0 and D7 (Fig. 3).

**Figure 5:**
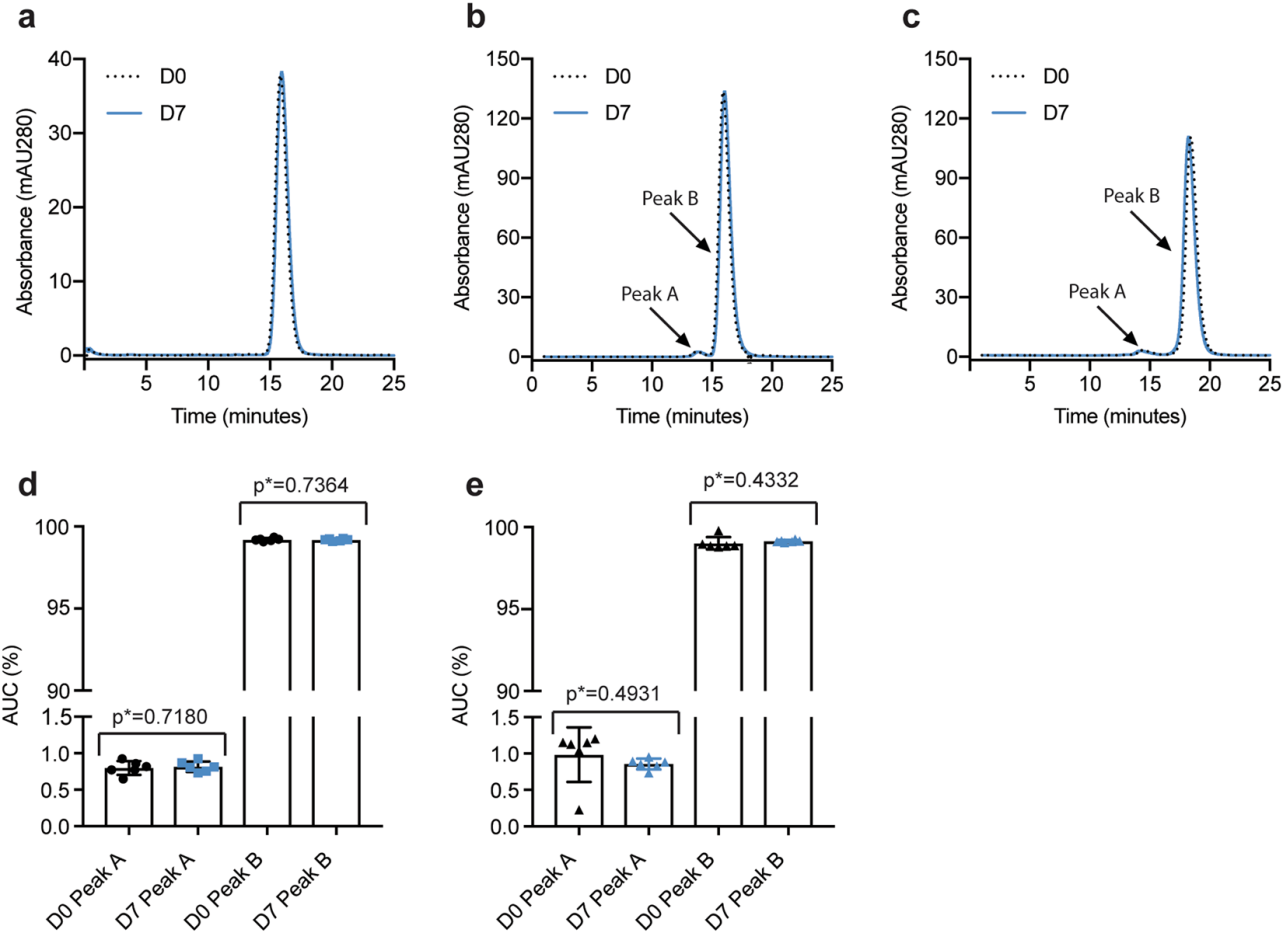
SEC analysis. SEC elution profiles of (**a**) ranibizumab, (**b**) aflibercept, and (**c**) bevacizumab at D0 (black) and D7 (blue). Comparison of AUC (%) of peak A and B for samples of (**d**) aflibercept and (**e**) bevacizumab from D0 and D7. Ranibizumab (**a**) only displayed one peak. The data are presented as mean ± SD. For each sample set n = 6. The unpaired Student’s t-test was used for statistical analysis.

Thirdly, to address whether compounding and storage caused non-covalent aggregation, size-exclusion chromatography (SEC) was performed using an ÄKTA avant 25. To avoid bias due to storage in the sample compartment, the D0 and D7 samples were run alternately. All three anti-VEGF biologics were eluted as one major peak for both D0 and D7 samples (Fig. 5a–c). Aflibercept and bevacizumab displayed an additional peak slightly before elution of the main fraction which was present in both D0 and D7 samples (Fig. 5d,e). There were no statistically significant differences between the two timepoints.

**Figure 6:**
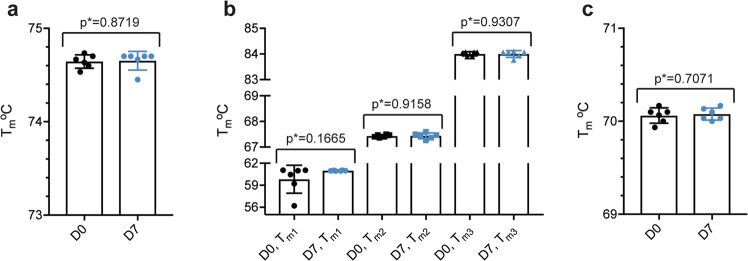
Thermal stability. Tm values for D0 (black) and D7 (blue) for (**a**) ranibizumab, (**b**) aflibercept and (**c**) bevacizumab. The melting process for (**a**) ranibizumab and (**c**) bevacizumab occurred in one event, whereas for (**b**) aflibercept, it occurred in three different temperature ranges. This was expected as aflibercept consists of three structurally different domains. The data are presented as mean ± SD. For each sample set n = 6, as measured in triplicates. The unpaired Student’s t-test was used for statistical analysis.

Finally, nano-differential scanning fluorimetry (nano-DSF) determining the melting temperatures of the compounded biologics was performed using a Prometheus NT.48. The results did not demonstrate significant differences between D0 and D7 for any of the biologics (Fig. 6).

### VEGF binding properties

**Figure 7:**
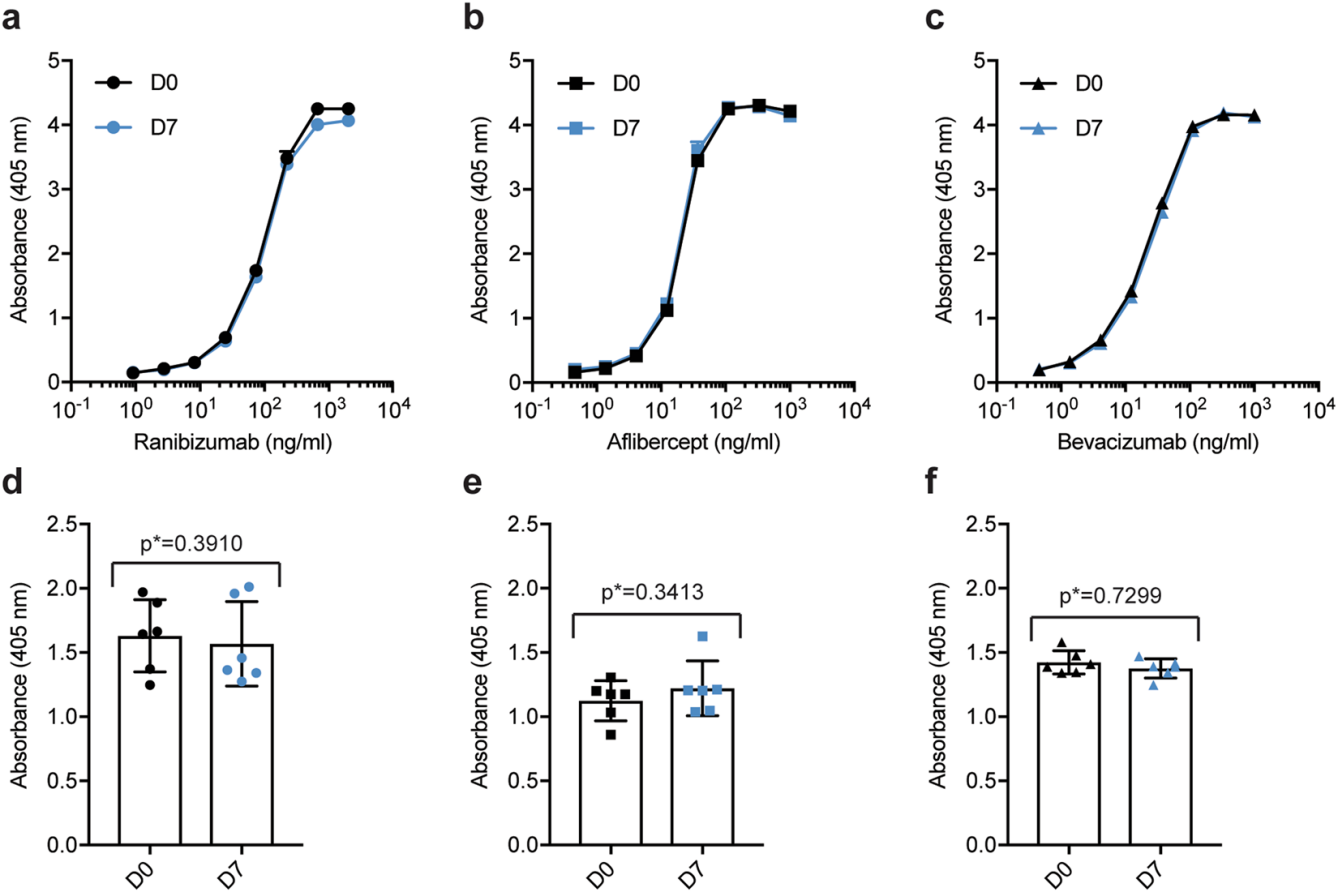
Binding properties to VEGF. Binding to VEGF in ELISA for titrated amounts (1000–0.5 ng/mL) of (**a**) ranibizumab, (**b**) aflibercept, and (**c**) bevacizumab at D0 (black) and D7 (blue). Comparison of binding to VEGF at D0 and D7 for the following concentrations: ranibizumab 74 ng/mL (**d**) and 12.34 ng/mL for aflibercept (**e**) and bevacizumab (**f**). For each sample set n = 6. The unpaired Student’s t-test was used for statistical analysis.

**Figure 8:**
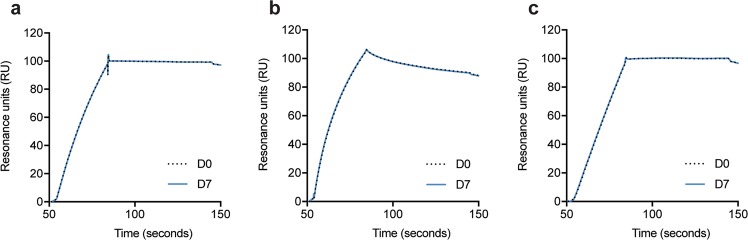
VEGF binding properties as measured by SPR. Sensorgrams showing the binding profiles to immobilized VEGF (~300 RU) for (**a**) ranibizumab (800 nM), (**b**) aflibercept (100 nM), and (**c**) bevacizumab (100 nM) at D0 (black dotted line) and D7 (blue). For each injection n = 6. The binding profiles (RU) have been normalized to baseline and the blank values subtracted.

The therapeutic effect of anti-VEGF biologics depends on their ability to neutralize soluble VEGF. To investigate how the compounded biologics bound VEGF, ELISA was performed. Plates were coated with a constant amount of recombinant human VEGF before adding titrated quantities of aflibercept, bevacizumab or ranibizumab. Bound biologics were visualized by adding a polyclonal anti-human IgG Fc antibody for aflibercept and bevacizumab and a polyclonal anti-human kappa light chain for ranibizumab. The analyses showed that the three biologics bound VEGF equally well at D0 and D7 (Fig. 7), as measurements retrieved from the exponential phases did not display significant differences (p = 0.7299 for ranibizumab, p = 0.3910 for aflibercept and p = 0.3413 for bevacizumab). These results were substantiated by SPR analysis where VEGF was immobilized and equal amounts of the three anti-VEGF biologics were injected. The resulting sensorgrams did not display differences in protein binding kinetics (Fig. 8), thereby confirming that compounding and storage does not affect the VEGF binding capacity of neither of the biologics.

### FcRn binding properties

**Figure 9:**
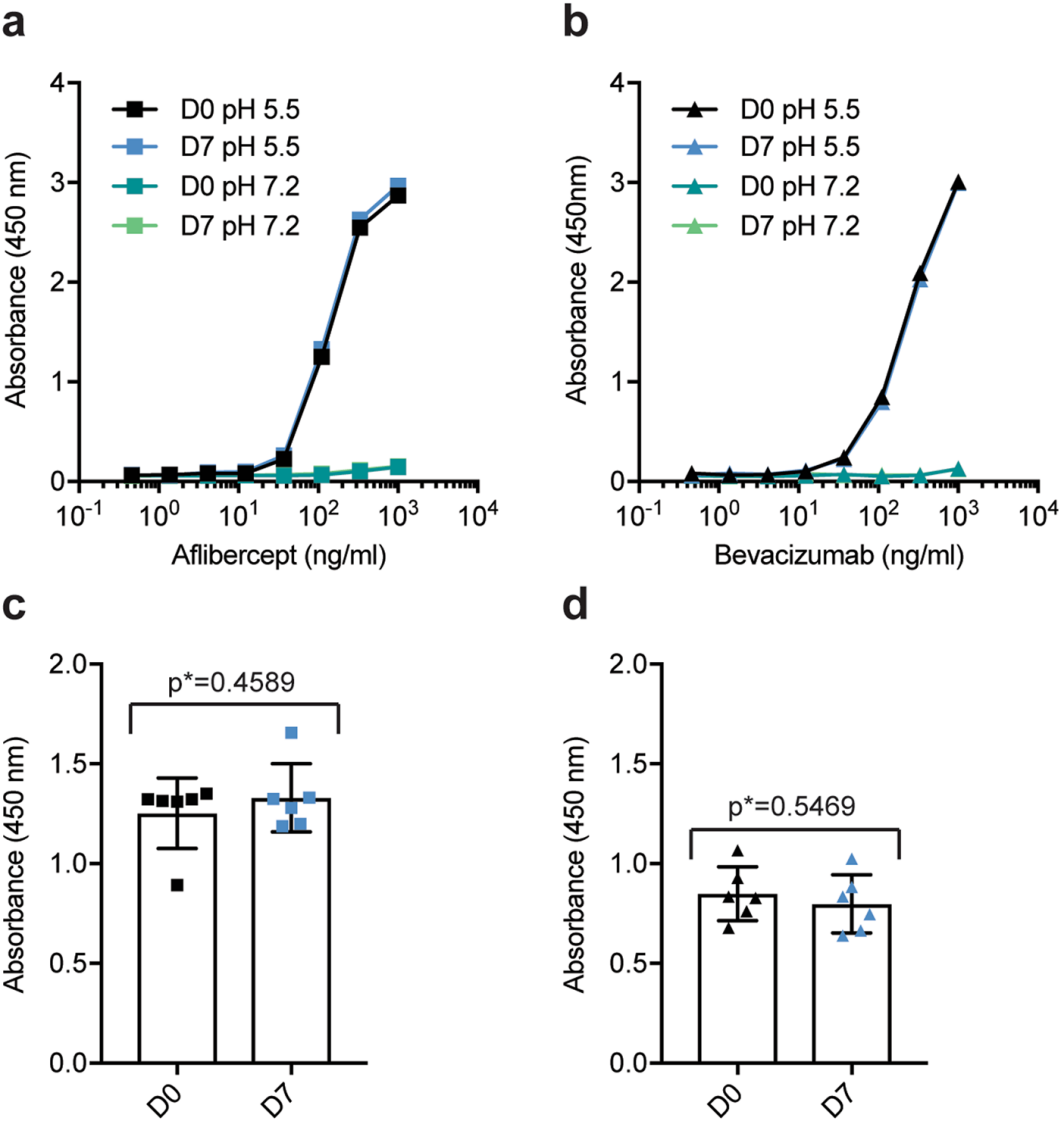
pH-dependent FcRn binding properties. Binding to a constant amount of GST-tagged human FcRn (1 µg/mL) at pH 5.5 and pH 7.2 for titrated amounts (1000–0.5 ng/mL) of (**a**) aflibercept and (**b**) bevacizumab at D0 (black and dark green) and D7 (blue and light green). Comparison of binding to human FcRn at D0 (black) and D7 (blue) at a given concentration of 111 ng/mL aflibercept (**c**) and bevacizumab (**d**). For each sample set n = 6. The unpaired Student’s t-test was used for statistical analysis.

An antibody recognizes a specific antigen epitope and may interact with effector molecules through its Fc domain. One such effector molecule, the neonatal Fc receptor (FcRn), is a key regulator of the long half-life of IgG in serum. FcRn additionally mediates bidirectional transport across cellular barriers, such as mucosal surfaces and the placenta^28–31^. Interestingly, the receptor is also expressed in ocular tissues and is involved in cellular uptake of IgG Fc-containing molecules and shuttling of intravitreally administered IgG across the blood-retinal barrier^32–36^. As such, we investigated the functional integrity of the Fc-containing anti-VEGF biologics, aflibercept and bevacizumab, by testing binding to human FcRn in ELISA. As the FcRn-IgG interaction is strictly pH dependent, strong binding at acidic pH 6.5-5.5 and no binding or release at neutral pH 7.4^29^, the assay was performed under both pH conditions. Titrated amounts of aflibercept or bevacizumab were added to plates coated with human VEGF, followed by a constant amount of GST-tagged human FcRn that was detected by an anti-GST antibody. The results showed that both aflibercept and bevacizumab bound equally well and in a pH dependent manner (Fig. 9a,b). Moreover, there were no statistically significant differences between D0 and D7 (Fig. 9c,d). Thus, pH dependent binding of human FcRn was not affected by storage in silicone oil-free syringes for 7 days.

## Discussion

Pharmaceutical compounding of anti-VEGF biologics for intravitreal injections can improve patient safety, save clinicians time, and reduce drug cost^10,20^. Prefilled syringes are commercially available for ranibizumab. Likewise, the U.S Food and Drug Administration (FDA) recently approved prefilled aflibercept syringes, which are anticipated in late 2019^37^. While the drug stability in these syringes presumably equals the findings in this study, one important difference remains: the present compounding procedure allows for in-house splitting of ranibizumab, aflibercept and bevacizumab vials under optimal hygienic conditions without compromising patient safety. Although prefilled syringes are available for two of the anti-VEGF biologics, the cost for one syringe is the same as for one vial. By comparison, the present procedure permits each vial to be divided into several syringes. Accordingly, the cost is reduced to almost one third for aflibercept, one half for ranibizumab and one fortieth for bevacizumab. Moreover, one compounding procedure can be standardized and safely implemented for all three intravitreally administered anti-VEGF biologics, ultimately avoiding splitting of the vials at the site of injection and thereby reducing risk for infections.

There is an increasing concern about symptomatic deposition of silicone oil droplets after repeated intravitreal injections^23–26^. We previously demonstrated that compounding and storage of aflibercept in silicone oil-coated insulin syringes had no negative effects on its biopharmaceutical properties^10^. The present study describes an important improvement of pharmaceutical compounding of the three most commonly used anti-VEGF biologics by utilizing silicone oil-free plastic syringes and thereby diminish the risk of symptomatic deposition of silicone oil droplets. As a silicone layer may prevent adsorption of the protein-based biologics to the plastic syringe’s internal surface, it is particularly important to address the possibility of drug-surface interactions for silicone oil-free syringes^11,16,17,38^. For that reason, we verified that the structural and functional properties were not altered before implementing the novel procedure into clinical practice. Seven days was chosen because it represents the maximum allowed storage time for aseptic magistral production according to Norwegian regulations. A thorough investigation of the biologics’ functional integrity did not reveal statistically significant differences between D0 and D7. The fact that neither VEGF nor FcRn binding properties were affected strongly supports the notion that drug efficacy is maintained throughout 7 days of storage.

Based on the results, pharmaceutical compounding of both aflibercept, bevacizumab, and ranibizumab in silicone oil-free prefilled plastic syringes is implemented as the standard of care for all intravitreal anti-VEGF injections performed in our hospital. The practice is also adopted in an increasing number of ophthalmic clinics, both in Norway and abroad.

One limitation of the study is that we have not investigated whether the biologics were affected by the withdrawal from the vial itself and into the syringe. Instead, newly drawn syringes were chosen as reference. Still, the chosen method is comparable to the clinical setting; drugs must be withdrawn from the vial in order to be injected into the eye, irrespective of whether the label recommendations or pharmaceutical compounding is utilized. A limitation of the current method itself is that the plunger resistance is altered^38,39^. For the 1.0 mL Injekt-F syringe used in the present study, there were only minimal dose variations. Yet, previous studies have shown highly variable accuracy and reproducibility when utilizing syringes that are commonly used for intravitreal injections^39–42^. Thus, a silicone oil-free syringe with a lower volume specifically manufactured for intravitreal use could further improve the current method.

In conclusion, we have established a novel method for pharmaceutical compounding of both bevacizumab, ranibizumab, and aflibercept for intravitreal administration, showing that silicone oil-free plastic syringes can be used without affecting the anti-VEGF biologics’ stability, molecular integrity or functional properties after 1 week of storage.

## Methods

### Repackaging process

Bevacizumab 25 mg/mL (Avastin; Roche), ranibizumab 10 mg/mL (Lucentis; Novartis) and aflibercept 40 mg/mL (Eylea; Bayer) were commercially acquired. Prefilled injection syringes intended for intravitreal injection were produced under standard aseptic conditions at the hospital pharmacy^10^. The syringes were filled according to the ISO 13544 guidelines and EU GMP^43,44^. The contents of the original vials were first withdrawn through a filter cannula (BD Blunt Fill Needle) into a 1 mL silicone free syringe (Injekt-F, 1 mL, B. Braun). The filter cannula was exchanged for a Low Dead Space Needle hub 33 G × 9 mm injection needle (TSK Laboratory) for aflibercept and ranibizumab, and 33 G × 13 mm needle for bevacizumab before approximately 0.06 mL was transferred to each of the ready-to-use syringe (Injekt-F, 1 mL). Each syringe was capped with a Low Dead Space Needle hub 33 G × 9 mm injection needle. The combined needle and syringe were separately enclosed in sterile, transparent plastic bags (Intervoid Sterile 250 mL; Coveris) and finally visually inspected and labelled outside the isolator. The syringes were stored in dark conditions at 4 °C for 7 days (D7). For dose accuracy testing at D7, a retina physician adjusted the volume of bevacizumab to 0.05 mL before fully depressing the plunger with the injection needle remaining attached. A second observer measured the weight of the syringes (n = 10) before and after the depression of the plunger. The volume was calculated based on a drug density (bevacizumab) of 1.0422 g/mL.

### Concentration measurements

The samples were transferred from prefilled syringes to sterile Eppendorf Protein LoBind-tubes (Eppendorf) that were kept on ice and protected from light during the experiments. All samples were diluted 1:10 in sterile phosphate buffered saline (PBS) (Sigma-Aldrich). Protein concentrations were measured using a DeNovix DS-11+ Spectrophotometer (DeNovix). Two measurements per sample were performed and the average values were calculated.

### SDS-page analyses

The protein samples were prepared by diluting 2 µg protein in distilled water and Bolt LDS loading buffer (Thermo Fisher Scientific), both with and without DL-dithiothreitol solution (Sigma-Aldrich). Samples containing DL-dithiothreitol solution were heated for 5 minutes at 95 °C. Next, the samples were applied to 12% Bolt Bis-Tris Plus gels (Invitrogen) before running for 22 minutes at 200 V. Spectra Multicolor Broad Range Protein Ladder (Fermentas) was used for size comparison, and the proteins were visualized by Bio-Safe Coomassie G-250 staining (Bio-Rad Laboratories).

### Size exclusion chromatography (SEC)

Aliquots were collected from the prefilled syringes and diluted in sterile PBS to the following concentrations: 5.6 mg/mL (aflibercept), 1.2 mg/mL (ranibizumab) and 3.5 mg/mL (bevacizumab). The experiments were performed using an ÄKTA avant 25 (GE Healthcare). Aflibercept and bevacizumab were run on a Superdex 200 Increase 10/300 GL column (GE Healthcare), whereas ranibizumab was run on a Superdex 75 Increase 10/300 GL column (GE Healthcare). For all samples, 77 μl was injected by means of an auto-sampler (Spark Holland B.V.).

### VEGF binding enzyme-linked immunosorbent assay (ELISA)

96-well EIA/RIA 3590 plates (Corning Costar) were coated with 100 μl 0.5 µg/mL human VEGF165 (Sino Biological) and incubated over night at 4 °C. The plates were blocked for 2 hours with 250 μl 4% skimmed milk powder (S) (Sigma-Aldrich) dissolved in PBS (Sigma-Aldrich) (S/PBS), followed by washing four times with PBS containing 0.05% Tween20 (T) (Sigma-Aldrich). Next, 100 μl titrated amounts (1000–0.5 ng for ranibizumab and 2000–0.9 ng for aflibercept and bevacizumab) of the anti-VEGF biologics diluted in S/PBS/T were added to the plates and incubated at room temperature (RT) for one hour on a shaker. After washing as previously described, 100 μl alkaline phosphatase (ALP)-conjugated goat anti-hFc Ab (Sigma-Aldrich) or ALP-conjugated anti-hKLC diluted to 1 μg/mL in S/PBS/T was added and incubated for 1 hour on a shaker. Following washing, the bound proteins were visualized by adding 100 μl ALP substrate (1 mg/mL) dissolved in diethanolamine buffer. The absorbance was measured at 405 nm using a Sunrise spectrophotometer (Tecan Group Ltd.).

### FcRn binding ELISA

96-well EIA/RIA 3590 plates (Corning Costar) were coated with human VEGF165 (Sino Biological) followed by blocking, before titrated amounts of the anti-VEGF biologics were added to the plates as previously described. Next, 100 μl of recombinant hFcRn-GST was added at a final concentration of 1 μg/mL diluted in S/PBS/T pH 5.5 (100 mM phosphate buffer, 0.15 M NaCl, 4% skimmed milk, 0.05% Tween 20) or S/PBS/T pH 7.4 and incubated for one hour at RT on a shaker^45^. After washing with either pH 5.5 or pH 7.4 PBS/T, horse radish peroxidase-conjugated anti-GST (Rockland Immunochemicals Inc) diluted 1:8000 in either pH 5.5 or pH 7.4 PBS/T was added and incubated for one hour at RT on a shaker. After washing as above, the bound receptor was visualized by adding 100 μl tetramethylbenzidine substrate (Calbiochem) followed by adding 100 μl 1 M HCl to stop the reaction. The absorbance was measured at 450 nm using a Sunrise spectrophotometer (Tecan Group Ltd.).

### Surface plasmon resonance (SPR)

A Biacore T200 (GE Healthcare) was used for measurements by immobilizing human VEGF165 (Sino Biological) (~300 resonance units (RU)) to CM5 sensor chips using amine-coupling as described by the manufacturer. The coupling was performed by injecting 5 µg/mL human VEGF165 dissolved in 10 mM sodium acetate pH 4.5 and using the amine coupling kit (GE Healthcare). HBS-P+ (0.01 M HEPES, 0.15 M NaCl, 0.005% surfactant P20, pH 7.4) was used as both running and dilution buffer. The measurements were performed by injecting 800 mM ranibizumab or 100 mM aflibercept and bevacizumab over the immobilized VEGF165 at a flow rate of 30 µl/min. Glycine at pH 1.5 (GE Healthcare) was used for regeneration of the CM5 chip between consecutive sample measurements. The sensorgrams were zero-adjusted and the individual injections normalized using the BIAevaluation software version 4.1 (GE Healthcare).

### Nano-differential scanning fluorimetry (DSF)

To determine the thermal stability of the anti-VEGF biologics, nano-DSF analysis was performed on a Prometheus NT.48 (NanoTemper Technologies GmbH). Undiluted samples were drawn into capillaries and run in triplicates. The instrument was set to gradually increase the temperature from 20 °C to 95 °C. As the temperature increased, the ratio between 330 nm and 350 nm wavelengths was plotted against temperature. The melting temperature (Tm) for which half of the proteins were unfolded was determined by deducing the first derivative in the PR.ThermControl software (NanoTemper Technologies GmbH).

### Statistical analyses

Figures were generated and the statistical analyses were performed using GraphPad Prism 7 (GraphPad Software). All antibodies were run in duplicates and tested at two separate occasions; error bars represent the SD from one representative experiment.

## Data Availability

The study’s datasets are available upon reasonable request to the corresponding author.
